# Supplementary material for: Metabolic and Transcriptomic Analyses Reveal the Effects of Ethephon on Taraxacum kok-saghyz Rodin
Source: Molecules. 2022 May 31;27(11):3548. doi: 10.3390/molecules27113548 (PMC9182187; doi:10.3390/molecules27113548)
Supplement: Supplementary file 1 [file molecules-27-03548-s001.zip › Supplementary Figure S1.pdf]

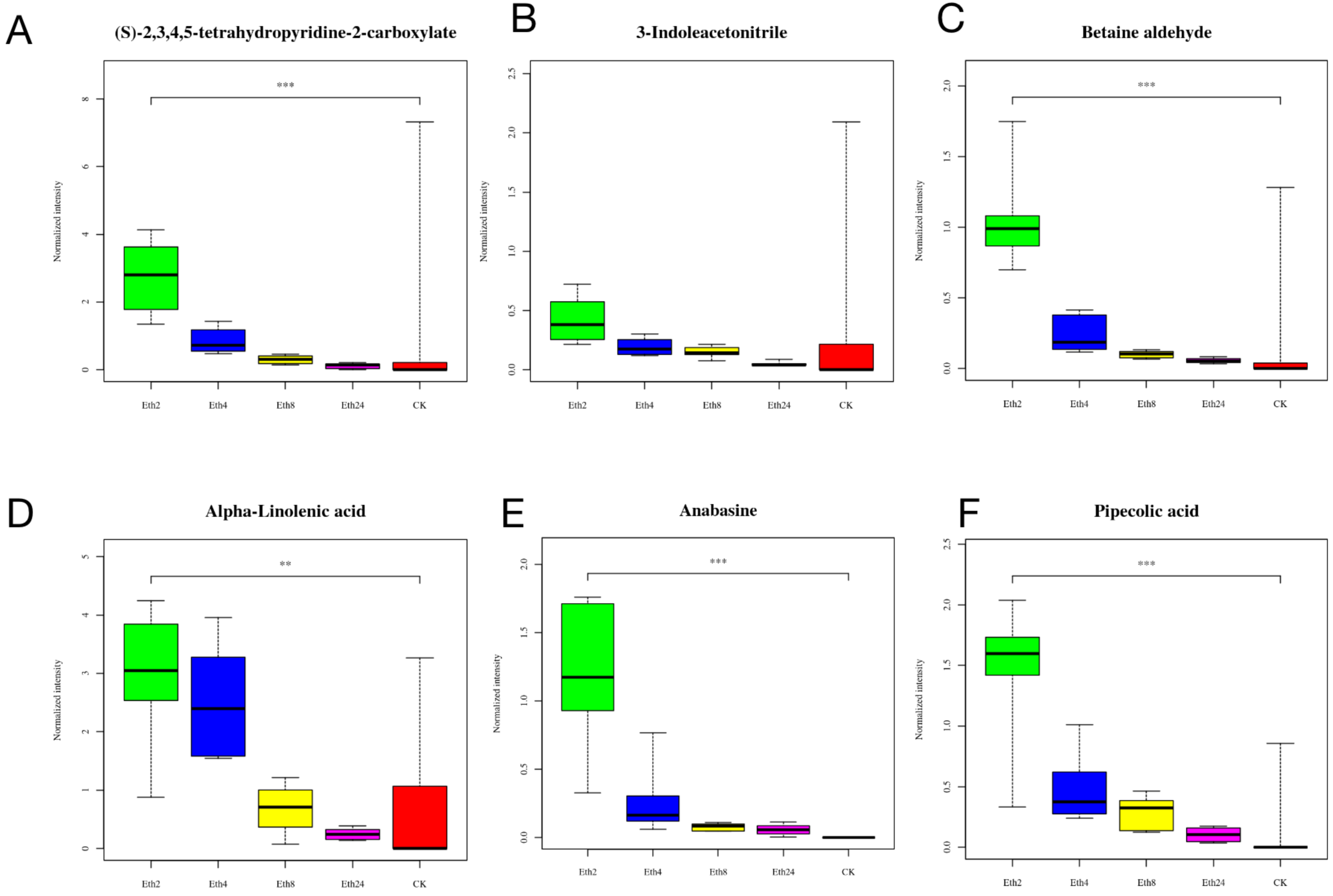
A
(S)-2,3,4,5-tetrahydropyridine-2-carboxylate
B
3-Indoleacetonitrile
C
Betaine aldehyde
D
Alpha-Linolenic acid
E
Anabasine
F
Pipecolic acid
Normalized intensity
Eth2
Eth4
Eth8
Eth24
CK
***
**
