# Supplementary figures and images for: Metabolic and Transcriptomic Analyses Reveal the Effects of Ethephon on Taraxacum kok-saghyz Rodin
Source: Molecules. 2022 May 31;27(11):3548. doi: 10.3390/molecules27113548 (PMC9182187; doi:10.3390/molecules27113548)

**A****3-(2-Hydroxyphenyl)propanoic acid**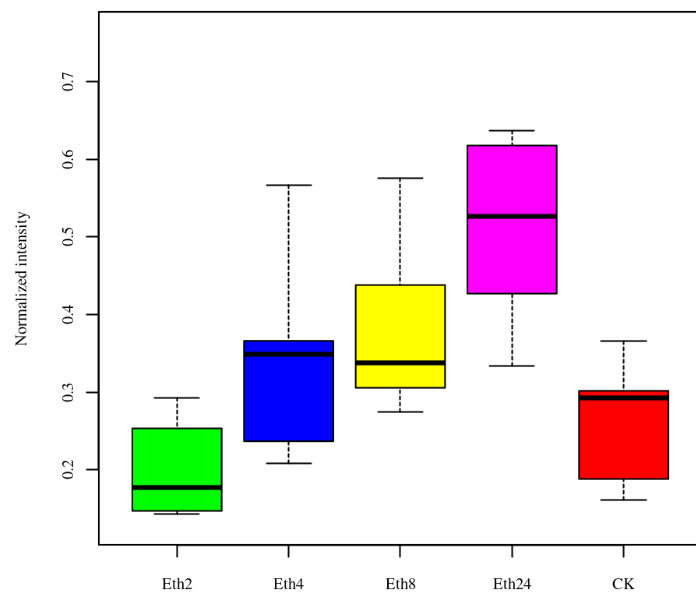**B****Bovinic acid**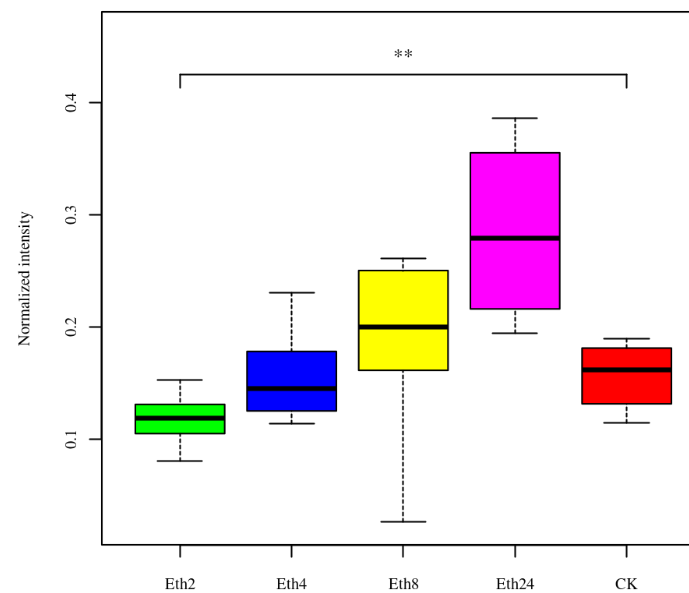**C****D-Galacturonate**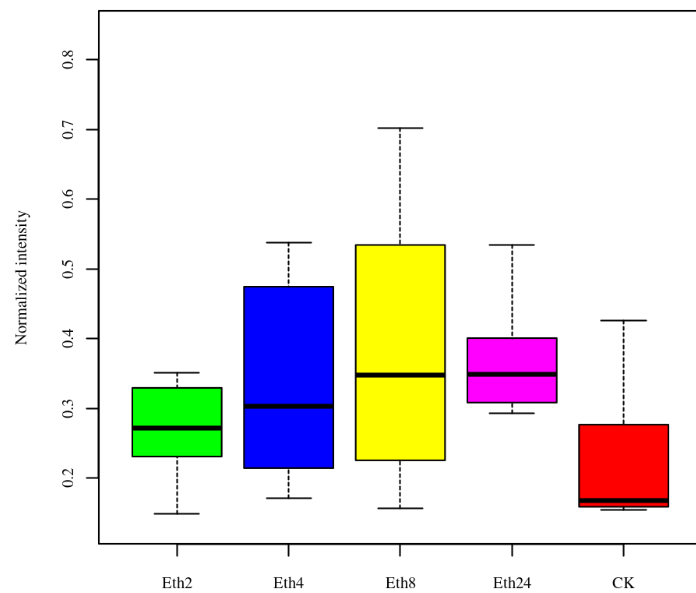**D****Gingerol**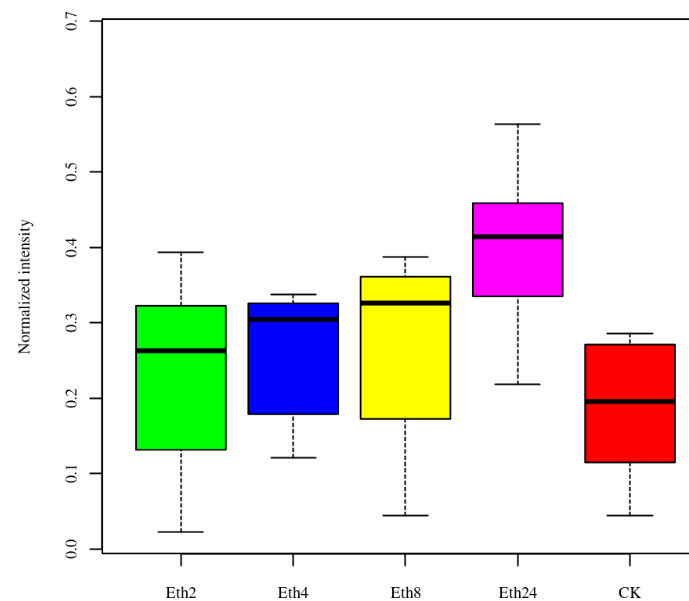

Supplement: Supplementary file 1 [file molecules-27-03548-s001.zip › Supplementary Figure S2.pdf]
